# Effects of wearable resistance load placement on neuromuscular activity and stride kinematics: A preliminary study

**DOI:** 10.17159/2078-516X/2022/v34i1a13102

**Published:** 2022-01-01

**Authors:** M Brown, C Giroux, M Lacome, C Leduc, K Hader, M Buchheit

**Affiliations:** 1 Paris Saint Germain, 5 Avenue du President John Fitzgerald Kennedy, Saint Germain-en-Laye, Paris, France, 78100; 2 French Institute of Sport (INSEP), Laboratory Sport, Expertise and Performance (EA 7370), Paris, France; 3 Kitman Labs, Performance Research Intelligence Initiative, Dublin, Ireland; 4 Carnegie Applied Rugby Research (CARR) centre, Carnegie School of Sport, Leeds Beckett University, Leeds, United Kingdom; 5 Playermaker, 35 Ballards Lane, London, United Kingdom N3 1XW; 6 Parma Calcio 1913, Performance and Analytics Department, Parma, Italy

**Keywords:** stride characteristics, electromyography, accelerometry, muscle activation

## Abstract

**Background:**

Wearable resistance (WR) training is a modality that allows athletes to perform loaded sport-specific movements to develop force and power outputs. The acute responses by which WR works is still relatively unknown, and the effects of WR load and location of the load has not yet been examined.

**Objectives:**

To investigate the acute neuromuscular and stride characteristic responses to different wearable resistance (WR) loads and placements on the calf muscles during high-speed running.

**Methods:**

Ten well-trained subjects completed a workout of ten sets of three 10s runs at 18km.h^−1^ (20s of rest between runs and one min between sets). Five conditions were tested: (1) unloaded control, (2) bilateral 0.75 vs. 1.5% body mass (BM) loading on the distal posterior calf, (3) bilateral proximal vs. distal loading of 1.5% BM positioned posteriorly, (4) bilateral anterior vs. posterior loading of 1.5% BM positioned distally, (5) unilateral loading of 1.5% BM on the distal posterior calf. Data were collected using Electromyography (EMG) and back-mounted GPS-embedded accelerometers. Magnitude of differences of within athlete and between muscle comparisons were calculated using effect sizes (ES) ± 90% confidence limits (CL).

**Results:**

No substantial differences in accelerometry data were observed between any of the loaded conditions and the control. EMG activity was lower for proximal loading compared to the control for the gluteus maximus (ES±90%CL; −0.72±0.41), vastus lateralis (−0.89±0.47) and vastus medialis (VM) (−0.97±0.46). Anterior loading induced substantially lower EMG activity for the semitendinosus (−0.70±0.48) and VM (−0.64±0.39) muscles compared with the control. EMG activity of the VM (−0.73±0.46) muscle was also substantially lower for posterior loading compared to the control. Unilateral loading induced no substantial differences in EMG activity between the loaded and unloaded legs.

**Conclusion:**

This preliminary study has provided a rationale for the performance of further investigations into the effects of WR lower limb loading on stride characteristics and EMG activity from a chronic standpoint using a larger population.

One of the keys for athletic performance is the successful transference of strength and power adaptations from gym-based exercises to sport-specific movements.^[1]^ A practitioner’s main goal when developing a resistance training programme is to develop an athlete’s strength and power outputs.^[2, 3]^ However, those improvements might not always transfer to on-field performance, with factors such as training methodology, volume, duration, and intensity influencing the transfer effect of training-induced strength and power adaptations.^[2, 3]^ To alleviate those inherent limitations, wearable resistance (WR) training may be a practical solution, with a better ecological fit than traditional gym-based resistance training. WR training allows athletes to perform loaded sport-specific movements which are hypothesised to provide greater transference to sport-specific movement performance compared with traditional gym-based exercises.^[1]^

WR has been used extensively within athletics and sprint training.^[1, 4]^ Research suggests WR can elicit increases in horizontal force production and improve sprint performance.^[1, 4]^ Additionally, investigations have explored the potential performance benefits of using WR for team sports.^[5–7]^ One study investigating the use of calf-loaded WR during warm-ups for elite footballers reported improvements in maximal horizontal (e.g. 10- and 20m sprints) but not vertical (e.g. counter movement jump) performance.^[5]^ In rugby players, the use of calf loaded WR (1% body mass, BM) resulted in a better maintenance of acceleration and sprint performance during a six week training period compared to unloaded players.^[6]^ Furthermore, WR training increased acute training load for high school American footballers loaded with 1% BM on the calves.^[7]^

In addition to WR training effects on locomotor performance, there is growing knowledge regarding movement kinematics and muscle coordination involved with WR loading.^[1, 8]^ During loaded sprinting, stride length and frequency decreased, while contact time and ground reaction forces increased.^[1]^ Previous research showed that the positioning of WR impacts stride kinematics.^[8])^ For example, greater kinematic changes were observed for calf loaded WR conditions compared to thigh loaded WR.^[1]^ However, it is yet to be investigated how the effect of WR varies based on lower limb load location (e.g. proximal vs distal placement, anterior vs posterior, bilateral/unilateral). If stride mechanics were to be altered, and/or specific muscle recruitment to be modified in relation to various WR placements (potentially affecting muscle coordination), this may have important implications for the integration of WR in training practices (e.g. rehabilitation).

Therefore, the aims of this preliminary study were to investigate changes in muscle activation amplitude and stride characteristics induced by the effects of WR calf loading of different loads (0.75%BM vs 1.5%BM), different load placements (anterior vs posterior and proximal vs distal), unilateral loading and loaded vs unloaded conditions during high-speed running efforts.

## Methods

### Subjects

Ten well-trained male subjects (30.9±6.0yrs, 178.6±5.4cm, 75.8±5.8kg), who regularly partake in running and resistance-based training (8 hours per week) completed this study. Table 1 shows the anthropometric data of the participants. Subjects were free from injury and illness for at least 4 weeks prior to the start and gave their consent for data obtained to be used in this study. Data collection was part of the club’s regular monitoring procedures which conformed to the Declaration of Helsinki.^(9)^

### Intervention

**Fig. 1:**
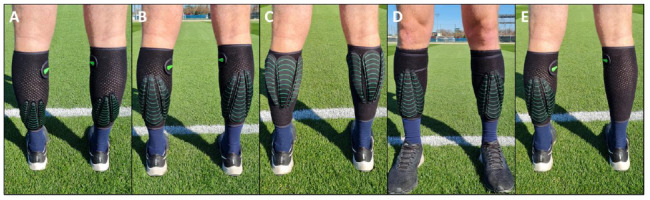
The experimental conditions – wearable resistance (WR) lower limb loading patterns. A) 0.75% BM distal, posterior loading; B) 1.5% BM distal, posterior loading; C) 1.5% BM proximal, posterior loading; D) 1.5% BM distal, anterior loading; E) 1.5% BM distal, posterior, unilateral loading.

Subjects completed a workout of ten sets of three 10s runs at 18km.h^−1^ with 20s rest between runs, and one min between sets. Each run was performed on a motorised treadmill (Skillrun Unity-7000, Technogym, Italy). Before each set, WR (Lila™ Exogen™, Malaysia) was placed on subject’s lower limbs. Five different WR conditions were tested: (1) control without load, (2) 0.75 vs 1.5% BM loading positioned on the distal posterior calf, (3) proximal vs distal loading of 1.5% BM positioned posteriorly (4) anterior vs posterior loading of 1.5% BM positioned distally, (5) unilateral loading of 1.5% BM positioned on the distal posterior calf (conditions 2–4 were bilaterally loaded). Figure 1 shows the experimental WR loading patterns. Data were collected using surface electromyography (EMG) and an embedded accelerometer within an upper back mounted GPS unit.

### Measurements

- Effect of load: 0.75% or 1.5% BM loading was placed on the posterior, distal portion of the lower limbs, aligned with the gastrocnemius aponeurosis line of action (Figs. 1A and B).
- Anterior/Posterior: To assess the effect of anterior/posterior loading, a 1.5% BM load was placed distally either on the front (anterior) or rear (posterior) of the lower limbs, aligned with the tibialis anterior insertion and the gastrocnemius aponeurosis line of action respectively (Figs. 1B and D).
- Proximal/Distal: To test the effect of proximal/distal loading, a 1.5% BM load was placed posteriorly either on the upper calf (proximal) or lower calf (distal) between the origins of the medial and lateral heads of the gastrocnemius and in alignment with the gastrocnemius aponeurosis respectively (Figs. 1B and C).
- Unilateral condition: The unilateral condition involved load placement on one leg with 1.5% BM placed on the posterior distal portion of the lower limb, aligned with the gastrocnemius aponeurosis, while the other leg was unloaded (Fig. 1E). Participants completed the unilateral trial with both legs and this data was pooled to give an average unilateral measure.
- Control condition: Running without additional load.

### Data collection procedures

#### Electromyography

A BTS FREEEMG 300 wireless surface EMG system (BTS® Quincy, USA) was used with sensors placed on the gluteus maximus (Gmax), bicep femoris (BF), semitendinosus (ST), rectus femoris (RF), vastus lateralis (VL) and vastus medialis (VM) muscles for each leg. The skin was shaved, gently abraded and cleaned with alcohol to minimise interelectrode impedance. The bipolar, silver/silver chloride, surface disc electrodes (Blue Sensor N-00-S/25; Ambu, Baltorpbakken, Denmark) were placed with a centre-to-centre distance of 2.5cm, longitudinally with respect to the underlying muscle fibre arrangement and located according to the Surface EMG for the Non-Invasive Assessment of Muscles (SENIAM) recommendations.

The sampling frequency was 1000Hz. The EMG data processing technique started with filtering the EMG signal (High pass, 15Hz, third order Butterworth filter). Secondly, the calculation of the muscle activity (mean root mean square, RMS) during each of the trials was performed. The first and last few steps were excluded from the recording window to keep only the stable phase of the run. The treadmill was continually moving with participants instructed to jump onto the side between trials. This minimised any acceleration required during each repetition in an aim to increase the stability of the runs. The mean RMS for each trial was calculated. The three trials for each condition were averaged (CV: 6.1–8.8%). The control condition (mean RMS) was used to normalise all other conditions. EMG data is displayed as a % of the normalised condition.

#### Stride characteristics

Embedded accelerometers (952 Hz) in GPS units (StatSports®, Northern Ireland) were used in indoor mode to calculate bilateral stride kinematics. Accelerometry raw data were further analysed using ADI software (Athletic Data Innovations, Sydney, Australia) to derive floor contact time (CT [seconds]), peak force (Newtons), stride frequency (steps/second), and vertical stiffness (Kvert [KN.m^−1^]).^(10)^ ADI-derived metrics were shown to be reliable with small to moderate error during high-speed running (standardised typical error: 0.52–0.67).^(10)^

#### Data analysis

Muscle activity and accelerometry data for bilaterally loaded conditions were calculated using the average of three repetitions per condition from both legs and normalised in relation to the control condition (%). Muscle activity for the unilateral condition used the pooled average from the loaded legs (average of loaded left and right legs) to compare with the unloaded legs (average of unloaded left and right legs) to observe possible changes in muscle activation.

### Statistical analysis

Data are presented as mean ± standard deviation (SD) and as effect size ± 90% confidence limits (CL). Data were first checked for normality (Shapiro-Wilks test). Log transformation was used when required to transform skewed data to approximately conform to normality. Data were back- transformed after analysis to return them back to original units. The athletes comparisons between muscles were made using effect sizes based on Cohen’s d principle to determine the magnitude of change between conditions (0.75 vs 1.5% BM, anterior vs posterior, proximal vs distal, unilateral loading, control compared with all conditions) using Hopkins scale: 0.2 (small), 0.6 (moderate), 1.2 (large), 2.0 (very large).^(11)^ When the CL of the effect size (ES) did not overlap the smallest worthwhile change (SWC) (0.2), the change was considered substantial and of the observed magnitude; if the CL overlapped the SWC, the change was unclear.^[12]^

## Results

**Fig. 2:**
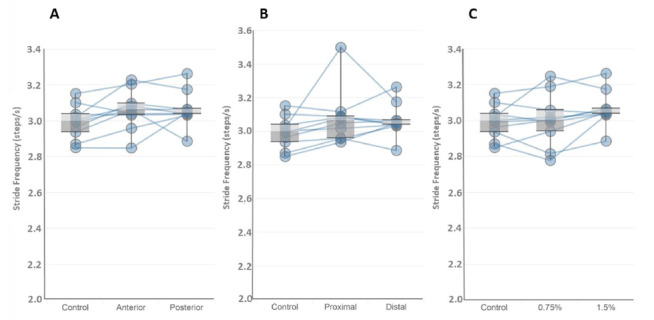
Intra-subject stride frequency (steps/sec) values for each wearable resistance (WR) load and loading pattern with group statistics shown within box plots. Each subject is represented by a line on each graph. A) Anterior and posterior loading compared to control, B) Proximal and distal loading compared to control, C) 0.75% and 1.5% BM loads compared to control.

**Fig. 3:**
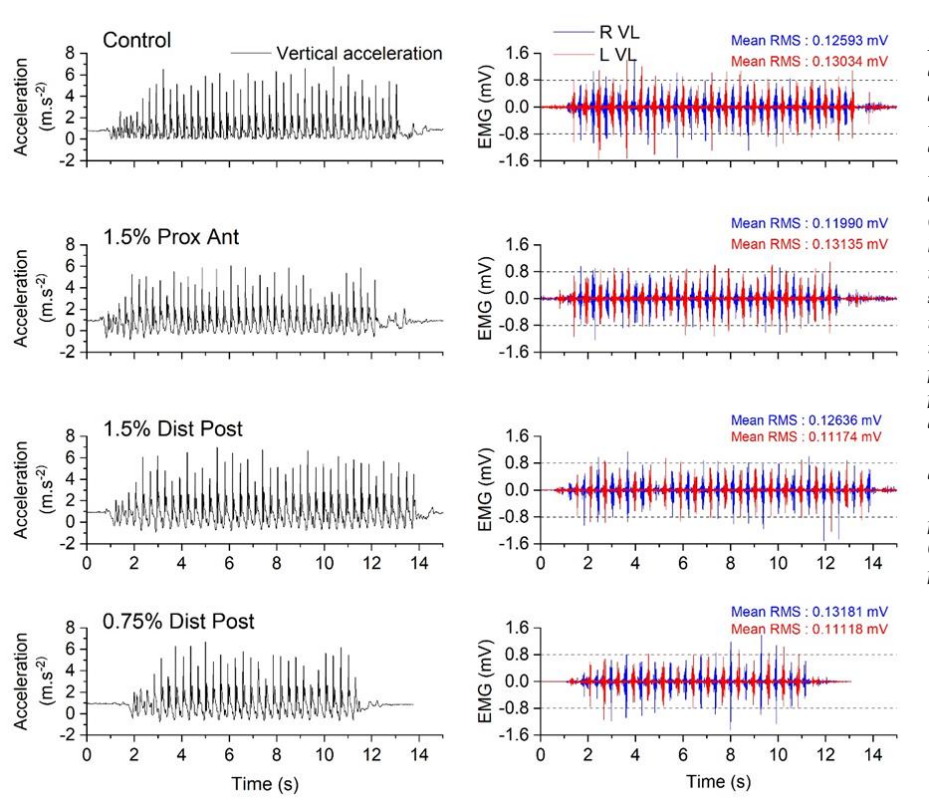
Typical raw data for vertical acceleration and EMG activity for four different conditions. EMG activity is expressed for the right (R) and left (L) vastus lateralis (VL). The mean root mean square (RMS) is indicated for each muscle and each trial presented. The four presented conditions are: control condition; 1.5% BM proximal, anterior loading; 1.5% BM distal, posterior loading; 0.75% BM distal, posterior loading.

Differences between conditions for accelerometry data are presented in Table 2 and differences between conditions for EMG data are presented in Table 3. Intra-subject variations between the stride frequency data is displayed in Figure 2 showing large individual responses to the load and placement. Figure 3 shows the synchronisation of left and right EMG data with vertical acceleration data for different loading patterns.

### Effect of load

No substantial differences were observed between 0.75% BM loading, 1.5% BM loading and the control for all stride characteristic metrics (all ES rated as unclear). Likewise, no substantial differences in stride characteristics were observed between 0.75% and 1.5% BM loading.

EMG activity for the VL and VM muscles were moderately lower for 0.75% BM loaded conditions compared with the control (ES:0.63–0.92). Additionally, 1.5% BM loading induced moderate decreases in EMG activity of the VM compared to the control (ES±90%CL; 0.70±0.44).

### Anterior vs posterior load placement

Accelerometry data showed no substantial differences for all stride characteristics of the anterior and posterior conditions compared to the control (all ES rated as unclear).

Anterior loading induced moderately lower EMG activity for the ST and VM muscles compared with the control (ST:0.70±0.48, VM:0.64±0.39). EMG activity of the VM muscle was also moderately lower for posterior loading compared to the control (0.73±0.46).

No substantial differences in stride frequency were observed between anterior and posterior loading. Furthermore, no substantial differences in EMG activity were observed for any muscle between anterior and posterior loading (all ES rated as unclear).

### Proximal vs distal load placement

Proximal and distal loading conditions showed no substantial differences in stride characteristics compared with the control. Moreover, accelerometry data showed no substantial differences in stride characteristics observed between proximal and distal loading (all ES rated as unclear).

The EMG activity of the Gmax, VL and VM was moderately lower for proximal loading compared to the control, while the EMG activity of the VM was moderately lower for distal loading compared to the control (ES:0.65–0.97).

### Unilateral loading

For unilaterally loaded conditions, no substantial differences in EMG activity were observed between the loaded and unloaded leg (all ES rated as unclear).

## Discussion

The aims of this preliminary study were to investigate the effects of different WR loads and loading placements during high-speed running efforts on stride characteristic variables and EMG responses. The main findings were as follows:

1. Bilateral WR loading induced no substantial changes in stride characteristics or force metrics for all loads and placements
2. Proximal loading patterns moderately decreased Gmax, VL and VM EMG activity, while distal loading patterns induced moderate decreases in VM EMG activity
3. Anterior and posterior WR load placement induced decreases in ST and VM EMG activity.

### Overall loading effects

Accelerometry data showed no substantial changes in stride characteristics (CT, stride frequency) and force metrics (peak force, Kvert) of the loaded conditions compared with the control. This study does not support previous findings regarding the effects of WR on stride characteristics and force metrics.^[1]^ Previous research highlighted that WR induced increases in stride frequency can occur in parallel to decreases in stride length,^[13]^ which may result in decreased lower limb muscle activity.^[14]^ However, as the observed changes in stride frequency were unsubstantial, changes in muscle activity may have been as a result of participants changing their joint kinematics, which may change the amount of muscle under the electrode thus potentially changing EMG amplitudes and recorded muscle activity.^[1]^ Regarding stride frequency, there were large differences in individual responses to the load and placements which are difficult to explain without EMG and accelerometry synchronisation but would be worth investigating in future studies. Previous studies have shown WR to increase the metabolic load of training.^[15]^ This, in addition to its ability to decrease neuromuscular load, may highlight the ability of WR to be a useful tool for coaches to utilise for training purposes while minimising injury risks. However, further research is necessary to quantify the effects of calf-loaded WR on stride kinematics.

### Effect of the load

When examining the effect of the load compared to the control, there were no substantial effects of 0.75% or 1.5% BM WR loading on stride characteristics or force metrics. Additionally, no differences were observed from the accelerometry data between the two loaded conditions. However, we found that EMG activity of the quadricep muscles (VL and VM) were substantially lower for the 0.75% BM loaded condition compared to the control. Furthermore, the VM muscle showed decreased EMG activity for the 1.5% BM condition compared to the control. The VL and VM are the hip flexors responsible for force production and the stabilisation of the knee during running ^[16, 17]^ and they exhibit their highest workload during the foot strike and early stance phases of running.^[16]^ These findings suggests that 0.75% and 1.5% BM loading induced decreases in the neuromuscular workload for the quadricep muscles responsible for force production during running without effecting running mechanics. Therefore, WR could potentially be a useful tool for rehabilitation protocols to reduce the neuromuscular load and potentially minimise injury risk. However, further research is required to investigate these findings using a larger testing cohort.

The finding of no substantial differences in accelerometry data between the two loaded conditions supports previous findings whereby there were no meaningful changes in stride frequency between 3% and 5% BM lower body loading.^[1]^ Contrary to the previous study^[1]^ in which the load was placed on the thighs and calves, the load in this study was focused solely on the calves. Calf loading can induce a greater rotational inertia than thigh loaded WR due to its increased distance from the rotational centre (hip joint) ^[1]^ thus, increases in calf loading could potentially result in greater effects to stride frequency than thigh loading. However, this needs to be investigated using a larger scale study. This information may be useful for coaches wanting to utilize WR loading patterns that may maximize performance adaptations elicited by this training modality. Interestingly, EMG data showed no changes in muscle activity between the 0.75% and 1.5% BM loaded conditions. This was potentially due to minimal increases in motor unit recruitment from the small loads relative to BM. Alternatively, in accordance with previous studies, the lack of EMG changes may have been due to a reduced EMG sensitivity to small differences in loads.^[18–20]^

### Effect of load placement

Regarding load placement, we found that anterior, posterior, proximal and distal load placements did not have any clear effects on stride characteristics. Previous research investigated the acute kinetic changes of shank vs thigh loading,^[1]^ but to our knowledge this is the first study to investigate varying calf loading patterns. There were no meaningful differences found between posterior and anterior 3% BM thigh loaded WR during sprint accelerations for kinematic measurements.^(1)^ However, we found that proximal loading caused decreases in Gmax, VL and VM EMG activity compared with the control and distal loading. Anterior and posterior loading also caused decreases in VM EMG activity, whereas no changes in stride frequency were observed between these conditions. The roles of the VL and VM muscles during running were previously stated and due to their importance for force production, proximally placed lower-limb WR loading may be a useful tool to reduce the neuromuscular workload of these muscles and reduce injury risks.^(16, 17)^ Moreover, due to their role as hip flexors involved in vertical movement patterns, such as raising the leg during running, the decreases in VL and VM EMG activity may suggest an association with decreases in stride length.^[17]^ However, further investigations are required to research these points in the context of WR training.

As previously stated, decreases in Gmax EMG activity was induced by proximal loading. The major functions of the gluteus maximus muscles during running are to provide trunk stability during the stance phase, decelerate the swing leg and assist with leg extension.^[21]^ The Gmax is most active during high-speed running.^[21]^ Decreases in Gmax EMG activity may be useful for reducing neuromuscular load during high-speed running thus proving to be a useful tool for rehabilitation training by reducing potential injury risks. However, as stated previously, further research is required to investigate these findings.

### Unilateral loading

Unilateral WR loading showed no substantial differences in EMG activity between the loaded and unloaded leg. As previously discussed, it is possible that the lack of substantial differences observed between the loaded and unloaded leg may have been due to a reduced EMG sensitivity to these small loads.^[18]^ Further research is required to assess the effects of WR unilateral loading on stride characteristics and EMG activity using greater loads.

While the study findings may not have been conclusive in identifying the stride characteristics and EMG activity of using lower-limb WR, the results from previous lower-limb loaded WR studies imply that using WR may allow coaches to induce a training overload explicit to sport-specific movement mechanics.^[5–7]^ Furthermore, lower-limb WR may be used to increase acute training workloads, but further research is required with larger sample sizes to understand the locomotor and neuromuscular effects of WR training in more detail.^[6, 7]^ Future research may also aim to increase the WR load or increase exposure times to the load, to investigate potential performance benefits of WR training. From a chronic standpoint, it is not known what performance adaptations a prolonged period of WR training could potentially provide, thus further research will be required to investigate this.

### Limitations

The main limitation of this study was the lack of mechanical and EMG data synchronisation which would allow the observation of EMG differences when different stride phases are used. While using a within activity normalisation approach for a high-speed, highly dynamic activity seems preferable, another approach which could have been used would be to perform maximal voluntary contractions (MVC) of each lower-limb muscle before testing and using this to normalise EMG signals across all conditions. This would have ensured accurate comparisons of intra- and inter-muscle activity. Furthermore, it was not possible to measure unilateral accelerometry data to identify this type of load placement effects on stride kinematics. Finally, the sample size of this preliminary study is small and thus lacks the statistical power to draw general conclusions. However, its value allows for the implementation of larger WR training studies with greater statistical power.

## Conclusion

This preliminary study suggests WR induces locomotor and neuromuscular changes during high-speed running. These findings have provided the rationale for the design of further research studies using larger sample sizes to investigate which stride characteristics and neuromuscular performance adaptations can be provided by acute and chronic exposure to WR training.

## Figures and Tables

**Table 1:** Individual subject anthropometric data: age, height and body mass

Subject	Age (yrs)	Height (cm)	Body mass (kg)
1	25.0	185	80.9
2	23.2	175	68.3
3	35.2	178	75.5
4	31.2	170	68.0
5	23.5	181	77.2
6	26.0	172	69.7
7	41.4	183	73.7
8	37.4	188	83.6
9	33.3	176	85.1
10	33.1	178	76.0
**Mean ± SD**	**30.9 ± 6.0**	**178.6 ± 5.4**	**75.8 ± 5.8**

**Table 2:** Accelerometry data: Bilaterally loaded conditions – 0.75% vs 1.5% body mass (BM) loading, Anterior vs Posterior loading, Proximal vs Distal loading

Accelerometry data	Mean ± SD			Effect Size ± 90% Confidence Limit		

	Control	0.75%	1.50%	Control vs 0.75%	Control vs 1.5%	0.75 vs 1.5%
**Contact Time (ms)**	0.17 ± 0.01	0.17 ± 0.01	0.17 ± 0.01	0.12 ± 0.28	0.11 ± 0.22	0.00 ± 0.20
**Peak Force (N)**	4 439 ± 299	4 448 ± 251	4 499 ± 232	0.04 ± 0.19	0.22 ± 0.24	0.18 ± 0.20
**Frequency (step/s)**	2.99 ± 0.10	3.01 ± 0.15	3.07 ± 0.10	0.08 ± 0.48	0.53 ± 0.44	0.45 ± 0.43
**kVert (KN.m** ^**−1**^ **)**	121 ± 14.9	120 ± 15.4	121 ± 15.9	−0.09 ± 0.33	0.00 ± 0.28	0.09 ± 0.17

	**Control**	**Anterior**	**Posterior**	**Control vs Anterior**	**Control vs Posterior**	**Anterior vs Posterior**

**Contact Time (ms)**	0.17 ± 0.01	0.17 ± 0.01	0.17 ± 0.01	0.27 ± 0.75	0.12 ± 0.22	−0.15 ± 0.95
**Peak Force (N)**	4 439 ± 299	4 492 ± 231	4 499 ± 232	0.38 ± 0.50	0.23 ± 0.24	−0.09 ± 0.47
**Frequency (step/s)**	2.99 ± 0.10	3.06 ± 0.12	3.07 ± 0.10	0.67 ± 0.50	0.59 ± 0.49	0.06 ± 0.78
**kVert (KN.m** ^**−1**^ **)**	121 ± 14.9	120 ± 16.3	121 ± 15.9	−0.50 ± 0.57	0.00 ± 0.27	0.07 ± 0.81

	**Control**	**Proximal**	**Distal**	**Control vs Proximal**	**Control vs Distal**	**Proximal vs Distal**

**Contact Time (ms)**	0.17 ± 0.01	0.17 ± 0.01	0.17 ± 0.01	0.17 ± 0.20	0.11 ± 0.22	−0.06 ± 0.18
**Peak Force (N)**	4 439 ± 299	4 531 ± 238	4 499 ± 232	0.34 ± 0.45	0.23 ± 0.24	−0.11 ± 0.45
**Frequency (step/s)**	2.99 ± 0.10	3.08 ± 0.17	3.07 ± 0.10	0.60 ± 0.64	0.52 ± 0.43	−0.08 ± 0.58
**kVert (KN.m** ^**−1**^ **)**	121 ± 14.9	122 ± 17.7	121 ± 15.9	0.02 ±0.24	0.00 ± 0.27	−0.01 ± 0.20

Frequency, stride frequency; kVert, vertical stiffness.

**Table 3:** EMG data: Bilaterally loaded conditions – 0.75% vs 1.5% body mass (BM) loading, Anterior vs Posterior loading, Proximal vs Distal loading

EMG data	% Normalised condition ± SD		Effect Size ± 90% Confidence Limit		

	0.75%	1.50%	0.75% vs 1.5%	Control vs 0.75%	Control vs 1.5%
**BF**	93 ± 36	93 ± 36	−0.04 ± 0.17	−0.34 ± 0.25	−0.38 ± 0.27
**Gmax**	71 ± 50	71 ± 50	0.01 ± 0.31	−0.50 ± 0.46	−0.50 ± 0.39
**RF**	83 ± 70	75 ± 67	−0.09 ± 0.24	−0.34 ± 0.29	−0.42 ± 0.33
**ST**	87 ± 31	80 ± 33	−0.16 ± 0.53	−0.47 ± 0.50	−0.63 ± 0.55
**VL**	75 ± 42	81 ± 39	0.11 ± 0.19	−0.63 ± 0.34*	−0.52 ± 0.41
**VM**	71 ± 50	77 ± 46	0.22 ± 0.18	−0.92 ± 0.40*	−0.70 ± 0.44*

	**Anterior**	**Posterior**	**Anterior vs Posterior**	**Control vs Anterior**	**Control vs Posterior**

**BF**	93 ± 36	93 ± 36	0.11 ± 0.15	−0.26 ± 0.30	−0.37 ± 0.26
**Gmax**	71 ± 50	71 ± 50	−0.13 ± 0.17	−0.39 ± 0.46	−0.52 ± 0.40
**RF**	92 ± 63	75 ± 67	−0.21 ± 0.26	−0.21 ± 0.30	−0.42 ± 0.33
**ST**	80 ± 25	80 ± 33	0.05 ± 0.28	−0.70 ± 0.48*	−0.65 ± 0.57
**VL**	81 ± 39	81 ± 39	−0.05 ± 0.16	−0.48 ± 0.35	−0.53 ± 0.42
**VM**	77 ± 46	77 ± 46	−0.08 ± 0.27	−0.64 ± 0.39*	−0.73 ± 0.46*

	**Proximal**	**Distal**	**Proximal vs Distal**	**Control vs Proximal**	**Control vs Distal**

**BF**	87 ± 39	93 ± 36	0.10 ± 0.25	−0.47 ± 0.31	−0.38 ± 0.27
**Gmax**	64 ± 44	71 ± 50	0.22 ± 0.36	−0.72 ± 0.41*	−0.50 ± 0.39
**RF**	75 ± 67	75 ± 67	−0.07 ± 0.22	−0.37 ± 0.30	−0.45 ± 0.35
**ST**	80 ± 33	80 ± 33	0.08 ± 0.39	−0.72 ± 0.59	−0.64 ± 0.56
**VL**	69 ± 45	81 ± 39	0.43 ± 0.29	−0.89 ± 0.47*	−0.46 ± 0.36
**VM**	65 ± 55	77 ± 46	0.32 ± 0.27	−0.97 ± 0.46*	−0.65 ± 0.41*

* represents a substantial difference compared with the other condition.

BF, bicep femoris; Gmax, gluteus maximus; RF, rectus femoris; ST, semitendinosus; VL, vastus lateralis; VM, vastus medialis.
